# 

**Published:** 1869-10

**Authors:** 


### Introductory Lecture Notice.

The lecture introductory to the regular course of lectures in the Buffalo Medical College, will be given Wednesday evening Nov. 3d, in the college ampitheatre 7½ o'clock, by Prof. JAMES P. WHITE. Members of the medical profession, and all ladies and gentlemen interested in the present *status* of the science of medicine and surgery are invited to be present.

J. F. MINER, Dean.

### Books and Pamphlets Received.

The Mechanism of Dislocation and Fracture of the Hip, with the reduction of the Dislocations by the Flexion Method. By Henry J. Bigelow, M. D, Professor of Surgery and Clinical Surgery in the Medical School of Harvard University; Surgeon to the Massachusetts General Hospital, etc. With illustrations. Philadelphia, H. C. Lea. For sale by T. Butler & Son.

The Pathology and Treatment of Stricture of the Urethra, and Urinary Fistulae. By Sir Henry Thompson, F. R. C. S.; Surgeon Extraordinary to the King of the Belgians. Professor of Clinical Surgery and Surgeon to University College Hospital. From the third and revised London edition. With Illustrations. Philadelphia, H. C. Lea. For sale by Breed & Lent.

A Handy Book of Ophthalmic Surgery for the use of Practitioners. By John Z. Laurence, F. R. C. S., M. D., (Univ. Lond.). Surgeon to the Ophthalmic Hospital, Southwark. Ophthalmic Surgeon to St. Bartholomews Hospital, (Chatham). Editor of the Ophthalmic Review, etc., etc. With Illustrations. Second American Edition, revised by the author, Philadelphia, H. C. Lea. For sale by T. Butler & Son.

Sleep and its Derangements. By Wm. A. Hammond, M. D. Professor of Diseases of the Mind and Nervous System, and of Clinical Medicine in the Bellevue Hospital Medical College; late Surgeon-General U. S. Army, etc., etc. Philadelphia, J. B. Lippincott & Co. For sale by Breed & Lent.

A Course of Practical Chemistry, arranged for the use of Medical Students. By Wm. Odling, M. B., F. R. S., Fellow of the Royal College of Physicians; lecturer on Chemistry at St. Bartholomews Hospital. With Illustrations. From the Fourth Revised London edition. Philadelphia, H. C. Lea. For sale by T. Butler & Son.

The Principles of Naval Staff Rank; with its history in the United States Navy for over half a century. By a Surgeon of the U. S. Navy. 1869.

Transactions of the Thirteenth Annual Meeting of the Illinois State Medical Society, held in Chicago in May, 1869.

Luxations of the Hip and Shoulder Joints, and the Agents which oppose their Reduction. By Moses Gunn, A. M., M. D. Professor of Surgery in Rush Medical College, Second Edition. Chicago, 1869.

Glaucoma. By Henry D. Noyes. M. D. Professor of Ophthalmology in Bellevue Hospital Medical College. Surgeon to the New York Eye and Ear Infirmary.

Electricity as a Means of Diagnosis, with a tabulated statement of five hundred cases treated mainly by General Electrization. By A. D. Rockwell, A. M., M. D. New York.

Printed Blanks for the Tabulated Statements of Hospitals for the Insane.

Rules for the course to be followed by bystanders in case of injury by Machinery or by Railroad. Prepared by John H. Packard, M. D., Secretary of the College of Physicians of Philadelphia.

Catalogue of the Medical Publications of Johannes Alt. Frankfurt an Main.

Hitchcock's Musical Magazine.

